# Sex differences in decision-making: Identifying multisensory behavioral differences in males and hermaphrodites

**DOI:** 10.17912/micropub.biology.000594

**Published:** 2022-07-29

**Authors:** Duncan Tanner, Denise Carigo, Chane Sevilla, Madison Lewis, Gareth Harris

**Affiliations:** 1 Biology Program, California State University Channel Islands, Camarillo, CA, USA

## Abstract

This present study uses *C. elegans* as a model to investigate how sex differences can influence sensory behavior and decision-making when encountering conflicting cues. We use a multi-sensory behavioral assay to characterize the differences between hermaphrodites and male worms when escaping from a food lawn during exposure to repulsive odors, such as, 2-nonanone. We find that male worms show a delayed food leaving during exposure to 2-nonanone when compared to hermaphrodite worms, and this is observed across multiple repulsive cues (2-nonanone and undiluted benzaldehyde) and multiple food types ( *E. coli* (OP50) and *Comamonas sp* ). Overall, this study provides a platform to further investigate how sensory-dependent decision-making behavior differs between sexes.

**Figure 1. Male and Hermaphrodite C. elegans show different food leaving behavior during exposure to undiluted repulsive odor cues:**
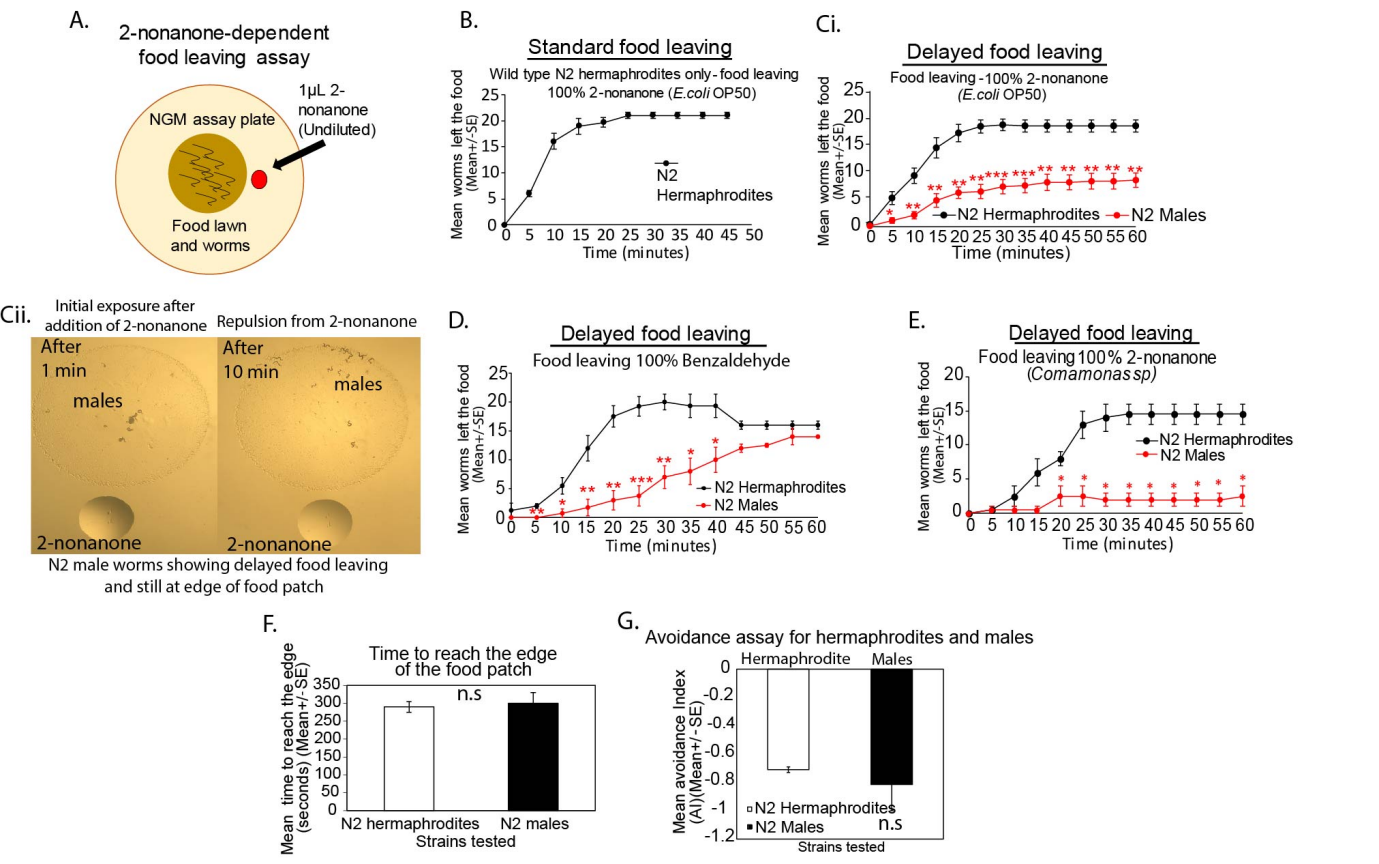
A-G **
A) Schematic of behavioral assay is shown that measures wild type hermaphrodite and male worms in a multi-sensory behavior. Assaying food leaving on *E. coli* (OP50) during exposure to undiluted 2-nonanone repellent** (See Methods, Harris *et al* ., 2019; Ellis *et al* ., 2020). All assays ranged from 45 - 60 minutes per assay for hermaphrodite worms being compared to male worms. **B)** **Wild type hermaphrodite worms show normal food leaving during exposure to undiluted 2-nonanone odor** (n=4 repeated days tested for wild type hermaphrodite worms) (0-45 minutes assayed). Wild type worms leaving food patch across 45 minutes (as described in Harris *et al* ., 2019; Ellis *et al* ., 2020). *E. coli* (OP50) food lawn used. 2-nonanone used. Mean number of worms that left the food at each 5 minute time point is represented, **C) i) Wild type N2 hermaphrodite worms versus wild type N2 male worms examined for 2-nonanone-dependent food leaving on *E. coli* (OP50)** (n=8 repeated days assayed for male vs hermaphrodite worms). (0 - 60 minutes assayed), males show delayed food leaving during 2-nonanone exposure, males were compared to hermaphrodite worms. Hermaphrodites, (Black line), males, (Red line), **ii)** Images showing wild type N2 male worms in the 2-nonanone-dependent food leaving assay (N2 males shown on *E. coli* (OP50) food patch just after 2-nonanone addition and after 10 minutes of 2-nonanone exposure (both images taken in the same experiment on the same day). **D) Males were examined for food leaving during exposure to the repellent, undiluted benzaldehyde (100%) compared to hermaphrodite worms** (0 - 60 minutes assayed), (n=4 repeated days assayed for male vs hermaphrodite worms), hermaphrodites, (Black line), males, (Red line). Delayed food leaving seen in males. *E. coli* (OP50) food lawn. Benzaldehyde used. **E) Wild type N2 hermaphrodite worms compared to N2 male worms were examined for 2-nonanone-dependent food leaving on a *Comamonas sp* food patch** (0 - 60 minutes assayed), (n=2 repeated days assayed for wild type male versus hermaphrodite worms), males were compared to hermaphrodite worms under identical conditions tested, hermaphrodites, (Black line), males, (Red line). Delayed food leaving seen in males. *Comamonas sp* bacterial food lawn used. 2-nonanone used. **F) Male worms were examined for their ability to reach the edge of the food patch during 2-nonanone exposure** (measuring 90% of worms that reach the edge of the food patch) (Harris *et al* ., 2019) (n=3 repeated days), n.s=not statistically significant when comparing hermaphrodite and male worms tested on same day. hermaphrodites, (White column), males, (Black column). **G)** **Wild type N2 male worms were examined for their ability to repel from 2-nonanone (100%, undiluted) when compared to wild type N2 hermaphrodite worms using the 2-nonanone avoidance assay** No food was present on the plate during this assay (See Methods, Troemel *et al* ., 1997). n=2 repeated days assayed for wild type hermaphrodites versus male worms. hermaphrodites, (White column), males, (Black column). Avoidance Index ranging from (0 to -1.0). We analyzed the Mean±SEM, Student’s *t* -test, * p ≤ 0.05, **p ≤ 0.01, ***p ≤ 0.001. All male worms were examined in parallel to wild type hermaphrodite worms tested on the same day under identical assay conditions. Each 2-nonanone-dependent food leaving assay for hermaphrodites or males used 20-25 worms per assay. n=number of repeated days tested for each behavioral assay from A-G.

## Description

Sex differences across organisms have been an intense area of research over the last 50 years. Despite these efforts, the mechanisms underlying many behavioral differences, including sensation, perception of sensory cues and decision-making and how this occurs at the level of the brain is still not understood. Sex differences have been identified from humans to worms. A number of studies have demonstrated differences between males and females in processes, including, olfaction, thermoregulation, aggression, learning and mood (Bjorkqvist *et al* ., 1994; Doty *et al* ., 1985; Berger-Sweeney *et al* ., 1995; Halpern *et al* ., 2000; Kaciuba-Uscilko *et al* ., 2001; Bielsky *et al* ., 2005). Behavioral studies in vertebrates and invertebrates have suggested that behavioral sex biases might have biological underpinnings. The understanding of the differences in neural signals and circuit function, that result in the variation in decision-making processes across female and male systems is not fully understood.

The hermaphrodite nematode *C. elegans* is attracted to or repelled by an array of volatile odorants (Bargmann and Horvitz, 1991; Troemel *et al* ., 1997; Debono and Maricq, 2005; Chao *et al* ., 2004; Zhang *et al* ., 2005; Ghosh *et al* ., 2017). In addition, hermaphrodites are able to generate decisions when encountering conflicting cues that are presented simultaneously (Ishihara *et al* ., 2002; Ghosh *et al* ., 2016; Harris *et al* ., 2019; Yang *et al* ., 2022). Male *C. elegans* are able to sense a number of chemicals, including, pheromones, food associated cues, diacetyl and salt (NaCl) (White *et al* ., 2007; Sakai *et al* ., 2013; Ryan *et al* ., 2014; Wexler *et al* ., 2020; Loxterkamp *et al* ., 2021; Portman *et al* ., 2017). To address sex specific differences in worm behavior, we used *C. elegans* to understand whether wild type hermaphrodites and males behave differently when exposed to conflicting cues, such as, food and the repulsive odor cue, 2-nonanone (Troemel *et al* ., 1997; Harris *et al* ., 2019; Ellis *et al* ., 2020).

We examined the behavioral differences between hermaphrodites and males using a “multi-sensory behavioral paradigm”, where we compare wild type *C. elegans* hermaphrodites and males in a food leaving assay when exposed to a repulsive cue, 2-nonanone (Fig. 1A, See schematic diagram, Harris *et al* ., 2019). We examined any differences between males and hermaphrodite adults in a multi-sensory behavioral assay, where both hermaphrodites and male worms were examined for food leaving during exposure to 2-nonanone. A number of genes and neurons have previously been identified for 2-nonanone-dependent food leaving in hermaphrodites (Harris *et al* ., 2019). Wild type hermaphrodites typically leave the *E.coli* (OP50) food patch within 10-15 minutes as normally observed after 2-nonanone addition next to the food patch (Fig. 1B and C). In contrast, when examining male worms we observe that males show a significantly delayed food-leaving during exposure to 2-nonanone (Fig. 1Ci (graph) and 1Cii (images)) showing an example of N2 males with reduced food leaving after 10 minutes). Suggesting, males leave a food patch significantly slower than hermaphrodites when stimulated by the presence of a repulsive odor. In addition, we observe that when N2 males leave the food patch during this period it is significantly delayed, and many of the males do not leave the food patch at all during our assay period of 45-60 minutes, despite positioning at the edge of the food patch furthest from the 2-nonanone (Fig. 1). Interestingly, wild type male worms have been shown to spontaneously leave a food patch, multiple hours later after being introduced to a food patch. But in the first hour of incubation of males on new food patch prior to adding 2-nonanone we do not see this increased leaving (Lipton *et al* ., 2004).

To address if N2 male worms were sensing 2-nonanone similar to hermaphrodites, or showing an actual difference in decision-making to leave the food, we used multiple approaches to do this. We analyzed male worm avoidance behavior to 2-nonanone using the 2-nonanone avoidance assay (Troemel *et al* ., 1997). We examine wild type males versus hermaphrodites in multiple ways. We found that N2 male worms avoid 2-nonanone the same as wild type N2 hermaphrodites (Fig. 1G). This was evident by N2 males avoiding 2-nonanone as strongly as wild type hermaphrodites tested in parallel. We also analyzed the male worms during the exposure to 2-nonanone while on the assay food patch. This was done by analyzing male behavior during the assay (See Fig. 1Cii Image of N2 males during repulsion) and measuring the time to reach the edge of the food patch after 2-nonanone was added next to the *E. coli* (OP50) food patch (see schematic diagram, Fig. 1a). Male worms crawled to the edge of the food and remained at the furthest edge away from the 2-nonanone repellent like wild-type hermaphrodite animals, suggesting N2 males could sense and perform a repulsive response to 2-nonanone (measured time taken for 90% of worms to reach the edge of the food) (Fig. 1F). This suggests that there is a difference in the decision to leave food during repulsive odor exposure, based on actual decision to leave food and not a difference in the general sensation of the repellent, 2-nonanone (Fig. 1F).

To further understand how general or specific these male differences to hermaphrodite worms are, we next varied two assay conditions. First, we used a second volatile repellent, benzaldehyde (undiluted) and measured benzaldehyde-dependent food leaving in males versus hermaphrodites (Yoshida *et al* ., 2012; Harris *et al* ., 2019). Interestingly, males also showed delayed food leaving in response to undiluted benzaldehyde when compared to wild type hermaphrodite animals (Fig. 1D). In addition, we changed the food patch type to assess any effect on male worm food leaving when compared to hermaphrodite worms (See Fig. 1C). Wild type hermaphrodite worms have been shown to exhibit a variety of behavioral outputs in response to food and food associated cues, including, showing varied feeding rates, sensory-dependent locomotory behavior, behavioral preference and food leaving dynamics (Zhang *et al* ., 2005; Shtonda and Avery, 2006; Meisel *et al* ., 2014; Olofsson *et al* ., 2014). We chose *Comamonas sp* food lawns and assessed male food leaving during exposure to 2-nonanone when compared to hermaphrodites *.* Wild type hermaphrodite worms also leave *Comamonas sp* during exposure to 2-nonanone, as previously described for wild-type hermaphrodites (Harris *et al* ., 2019; Ellis *et al* ., 2020). Upon examining male worms for 2-nonanone-dependent food leaving when compared to hermaphrodite worms, male worms are also significantly delayed food leavers on *Comamonas sp* , suggesting male worms show this general delayed food leaving across multiple foods, including at least, the regular tested food source, *E. coli* (OP50) and also *Comamonas sp* during exposure to 2-nonanone (Fig. 1E).

Overall, this suggests that wild type N2 male worms show decreased food leaving when compared to N2 hermaphrodites when exposed to different volatile repellents, including, undiluted 2-nonanone and benzaldehyde, and across different food types. Wild type hermaphrodites and males are known to behave differently in specific odor-guided behavior, locomotory dynamics and conditioning associated with salt chemotaxis (Loxterkamp *et al* ., 2021; Sakai *et al.* , 2013). This provides an avenue for further investigation of the neural mechanisms that influence behavioral differences observed between sexes in a multi sensory behavior.

## Methods

*C. elegans* strains were cultivated under the standard conditions (Brenner *et al* ., 1974). Wild type N2 hermaphrodite young adults, and wild type male adults were used in the 2-nonanone-dependent food leaving experiment for this study.

**Worm culturing, plate preparation and multi-sensory assay.** All hermaphrodite and male worms were grown to young adult under *E. coli (* OP50) well-fed conditions prior to performing behavior assay (Brenner *et al* ., 1974; Harris *et al* ., 2019).

**Multi-sensory assay to analyze male vs hermaphrodite adult worms.** For the 2-nonanone-dependent food leaving assay. Wild Type worms (N2) were assayed as previously described (Harris *et al* ., 2019; Ellis *et al* ., 2020). For all worms, *E. coli* (OP50) was used as the food source (Brenner *et al* ., 1974). For the preparation of assay plates, the NGM (Nematode Growth Medium) agar was made and poured into small assay plates (6 cm) and then allowed to cool and solidify (using standard NGM plate preparation protocols). After two days, a liquid suspension of *E. coli* (OP50) was prepared; consisting of 40 mL of NGM media and *E. coli* (OP50) colonies added to the NGM media, this media was then placed at 26°C overnight. The next morning, the *E. coli* (OP50) culture was centrifuged at 3500 rpm for 15 min. 35 mL of the NGM was removed and discarded (Harris *et al* ., 2019; Ellis *et al* ., 2020). The *E. coli* (OP50) pellet was then re-suspended in 5 mL of NGM through mixing, and then 55 µL of this *E. coli* (OP50) culture was then added to the center of an NGM agar assay plate (as seen in Fig. 1A, Schematic of behavioral assay). All worms were grown at 20-23°C on NGM plates that contained a lawn of *E. coli* (OP50). Next 20-25 young adult worms (either hermaphrodite or male worms) are placed onto the 1 cm diameter food lawn and are allowed to acclimate for 60 minutes. After 1 hour, a drop of 2-nonanone repellent (1 µL) is placed approximately two worm lengths away from the food lawn. The number of worms on the lawn was counted every 5 minutes for a total of 45-60 minutes, and the number of hermaphrodite or male worms that left the food lawn was determined. Food leaving is analyzed during exposure to the repellent 2-nonanone (Harris *et al* ., 2019; Ellis *et al* ., 2020). Worms present on the food at each 5-minute time-point are counted (represented in y-axis). For each plate of male worms tested, there was a separate hermaphrodite assay plate examined in parallel. No males were mixed with hermaphrodite adult worms during the behavioral assay. For all 2-nonanone-dependent food leaving assays on *Comamonas sp* food lawn, the assay was prepared and tested using identical conditions to *E. coli* (OP50) assays tested above. For all data analysis, a *student’s t test* was performed when comparing wild type N2 adult hermaphrodite worms to wild type N2 male adult worms tested on the same day in parallel conditions. Mean±SEM, Student’s *t-* test, *p ≤0.05, **p≤0.01, ***p≤0.001. n = number of repeated days tested across 1A - G.

**Assay to measure the ability to reach the edge of a food patch during 2-nonanone exposure**

Wild type hermaphrodites and male worms were examined for their ability (Time to reach the edge) to reach the furthest edge of the food patch during exposure to undiluted 2-nonanone. 20-25 hermaphrodites and males were examined per replicate. Wild type N2 hermaphrodites and wild type N2 males were examined for the time taken for 90% of the worms to reach the edge of the food patch furthest from the 2-nonanone drop placement (last sector of the food patch). Time was determined as time taken (sec) for worms to reach the edge of the food from the point of 2-nonanone addition next to the *E. coli* (OP50) food patch. The 1 cm food patch was divided into 5 sectors (0.2 cm each). Sectors A on food patch being closest to the 2-nonanone, Sector E on food patch being furthest away from 2-nonanone drop (As previously described in Harris *et al* ., 2019). Wild type N2 male worms were measured and compared to wild type N2 hermaphrodites tested in parallel on separate behavior assay plates. Wild type N2 hermaphrodite and male worms were not mixed on behavioral assay plates. Mean±SEM, student’s *t-t* est, *p≤0.05, **p≤0.01, ***p ≤0.001. n=number of repeated days tested for hermaphrodite and male worms.

**2-nonanone avoidance assay**

To examine the avoidance of 2-nonanone in hermaphrodites and males, chemotaxis assays were performed essentially as previously described (Troemel *et al* ., 1997; Harris *et al* ., 2019). Briefly, animals were placed in the center of a square plate that was divided into sectors A, B, C, D, E and F and 2 drops of 1 µl of undiluted 2-nonanone was added to one side and 2 drops of 1 µl ethanol was added to the opposite side of the plate as a control. Approximately 75-100 hermaphrodite or male worms were used in each assay. 2-nonanone avoidance was analyzed by counting the number of worms in the sectors A-B, C-D, and E-F with E-F being furthest away from the 2-nonanone point sources. The avoidance index was calculated as the number of animals in sectors A and B (at 2-nonanone) minus the number of animals in the sectors E and F (at control) and normalized with the total number of animals in all 6 sectors on plate (Avoidance Index between 0 and -1.0). For all data analysis, *a Student’s t test* was performed when comparing wild type N2 hermaphrodite worms to wild type N2 male worms tested on the same day in parallel identical conditions. Mean±SEM, Student’s *t* -test, p ≤0.05*, p≤0.01**, p≤0.001***.

## Reagents

**List of worm and bacterial strains used in experiments in the present study**

1) Wild type N2 Bristol hermaphrodite *C. elegans* were purchased from CGC,

2) N2 wild type male *C. elegans* were purchased from CGC,

3) *E. coli* (OP50) bacterial strain was purchased from CGC,

4) DA1877 *Comamonas sp* bacterial strain was purchased from CGC,

All strains were provided by the CGC (Caenorhabditis Genetics Center) at the University of Minnesota, which is funded by NIH Office of Research Infrastructure Programs (P40 OD010440).

This project was funded through lab start-up (Harris G), California State University Channel Islands and Mini-Grant awarded, California State University Channel Islands, 2020 - 2021.

**Chemicals used in study.** All chemicals used in this study, including, 2-nonanone (Cas # 821-55-6) and Benzaldehyde (Cas # 100-52-7) were purchased from Sigma Aldrich.
